# Pronounced Peramorphosis in Lissamphibians—*Aviturus exsecratus* (Urodela, Cryptobranchidae) from the Paleocene–Eocene Thermal Maximum of Mongolia

**DOI:** 10.1371/journal.pone.0040665

**Published:** 2012-09-19

**Authors:** Davit Vasilyan, Madelaine Böhme

**Affiliations:** 1 Department for Geoscience, Eberhard-Karls-University Tübingen, Tübingen, Germany; 2 Senckenberg Center for Human Evolution and Palaeoecology (HEP), Tübingen, Germany; Oxford Brookes University, United Kingdom

## Abstract

**Background:**

The oldest and largest member of giant salamanders (Cryptobranchidae) *Aviturus exsecratus* appears in the latest Paleocene (near the Paleocene–Eocene Thermal Maximum) of Mongolia. Based on femoral and vertebral morphology and metrics, a terrestrial adaptation has been supposed for this species.

**Methodology/Principal Findings:**

A detailed morphological reinvestigation of published as well as unpublished material reveals that this salamander shows a vomerine dentition that is posteriorly shifted and arranged in a zigzag pattern, a strongly developed olfactory region within the cranial cavity, and the highest bone ossification and relatively longest femur among all fossil and recent cryptobranchids.

**Conclusions/Significance:**

The presence of these characteristics indicates a peramorphic developmental pattern for *Aviturus exsecratus*. Our results from *Av. exsecratus* indicate for the first time pronounced peramorphosis within a crown-group lissamphibian. *Av. exsecratus* represents a new developmental trajectory within both fossil and recent lissamphibian clades characterized by extended ontogeny and large body size, resembling the pattern known from late Paleozoic eryopines. Moreover, *Av. exsecratus* is not only a cryptobranchid with distinctive peramorphic characters, but also the first giant salamander with partially terrestrial (amphibious) lifestyle. The morphology of the vomers and dentaries suggests the ability of both underwater and terrestrial feeding.

## Introduction

The recent species of the clade Cryptobranchidae are characterized by unicapitate rips, huge body size, obligate paedomorphy, and strict aquatic lifestyle. The adult vomerine teeth are of larval dentition type, i.e., lying in a curved row parallel to the maxillary dentition. The lacrimal and septomatomaxillary bones as well as the eyelids are absent [1]–[5]. In the present day, one species of giant salamander inhabits North America, China and Japan, respectively [3]. It is accepted that fossil cryptobranchids do not differ significantly from the morphology and biology of living forms [2], [4], [6], [7], although terrestrial adaptations were suggested for *Av. exsecratus* based on the some peculiarities of the axial skeleton, femur, and skull [8].

*Aviturus exsecratus* is the oldest Cenozoic giant salamander species from Eurasia. It was described by Gubin [8] from the terminal Paleocene Nara-Bulak formation of Mongolia (Paleocene-Eocene Thermal Maximum).

Here we re-examine the material described by Gubin [8] and study additional bones of *Av. exsecratus* from the Naran-Bulak formation. Our results substantiate the suggestion of Gubin [8] that this species exhibit terrestrial adaptations. Moreover, we provide extended information on life style and life history strategy by analyzing skull morphology, the vomerine dentition, the olfactory region of cranial cavity, bone density, as well as the axial and appendicular skeleton. Importantly, we show that *Av. exsecratus* is the first lissamphibian showing a peramorphic life history. Based on our results we are in the position to emend the diagnosis of the family Cryptobranchidae.

## Results

### Systematic palaeontology

**Amphibia Linnaeus, 1758.**

**Lissamphibia Haeckel, 1866 (sensu Pyron, 2011).**

**Caudata Scopoli, 1777.**

**Urodela Duméril, 1806.**

**Cryptobranchidae Fitzinger, 1826.**

#### Type species

*Andrias scheuchzeri* (Holl, 1831).

#### Distribution

Possibly in the Late Jurassic of China; Paleocene-Holocene of the Holarctic.

#### Emended diagnosis

Very large salamanders up to 2 meters, with paedomorphic or peramorphic life history strategy, and aquatic or amphibious life style. Bilateral asymmetric kinetics of the lower jaw. Parietal and squamosal bones are directly connected and ribs are unicapitate.

***Aviturus*** **Gubin, 1991.**

#### Type species

*Aviturus exsecratus* Gubin, 1991.

#### Distribution

As for the type and only species.

#### Revised Diagnosis

Same as for the type species and only species.

***Aviturus exsecratus*** **Gubin, 1991.**

**Figure 1:**
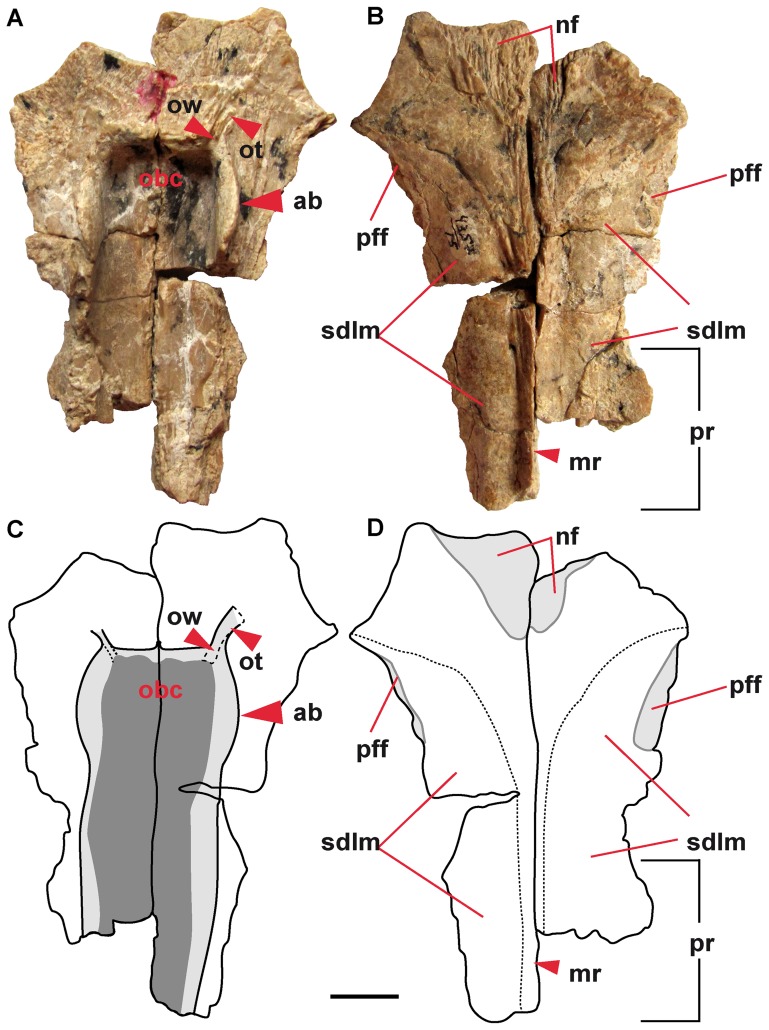
Frontal (PIN 4357/15) of *Aviturus exsecratus* from ventral (A, C) and dorsal (B, D) views. (A), (B) photographs and (C), (D) graphic representations. **Abbreviations**: ab, anterior bump; mr, medial (sagittal) ridge; nf, nasal facet; pff, prefrontal facet; pr, parietal ramus; obc, olfactory region of cranial cavity; ot, olfactory tract; ow, olfactory windows; sdlm, attachment surface of deep levator mandibulae anterior muscle. Scale bar = 1 cm.

**Figure 2:**
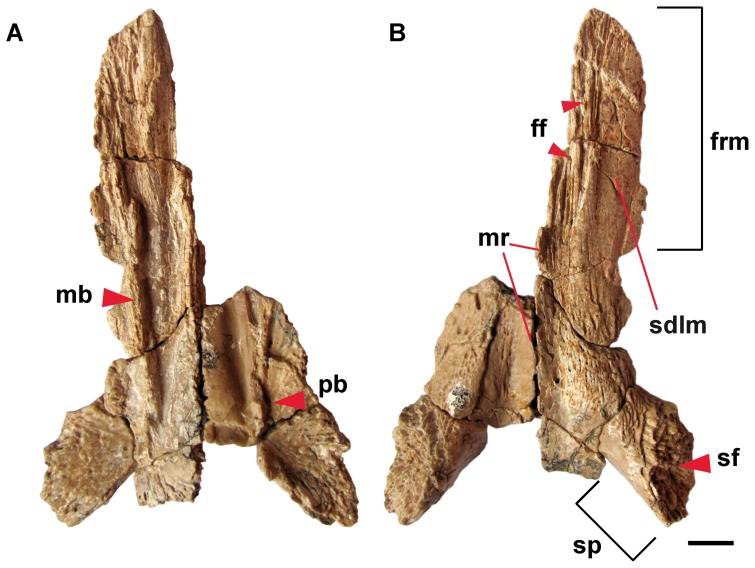
Parietal (PIN 4357/13+73) of *Aviturus exsecratus* in (A) ventral and (B) dorsal views. **Abbreviations**: ff, frontal facet; frm, frontal ramus; mb, medial bump; mr, medial (sagittal) ridge; pb, posterior bump; sdlm, attachment surface of deep levator mandibulae anterior muscle; sf, squamosal facet; sp, squamosal process. Scale bar = 1 cm.

**Figure 3:**
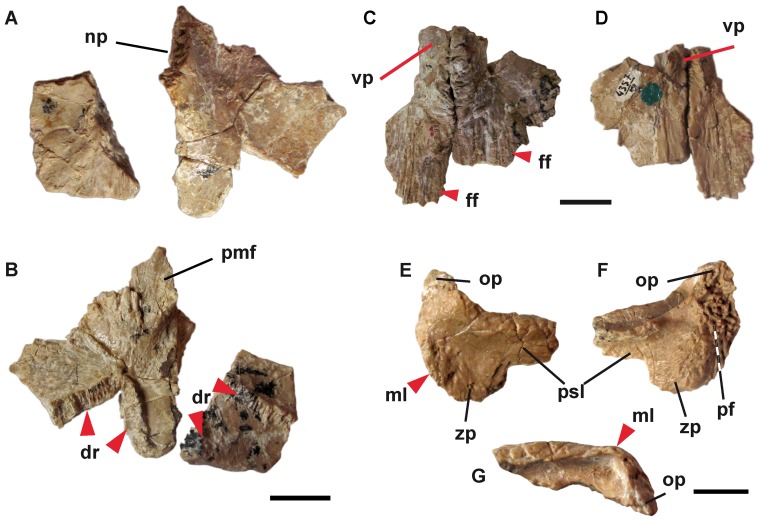
Right and left vomers (PIN 4357/13), nasals (PIN 4357/7), and right squamosal (PIN 4357/73) of *Aviturus exsecratus*. (A) dorsal and (B) ventral views of vomers, (C) ventral and (D) dorsal views of nasals and (E) dorsal, (F) ventral, (G) anterior views of right squamosal. **Abbreviations**: dr, dental row; ff, frontal facet; ml, marginal lamina; np, nasal process; op, otic process; pf, parietal facet; psl, posterior squamosal lamina; pmf, premaxillar facet; vp, vomerine process; zp, zygomatic process. Scale bar = 1 cm.

**Figure 4:**
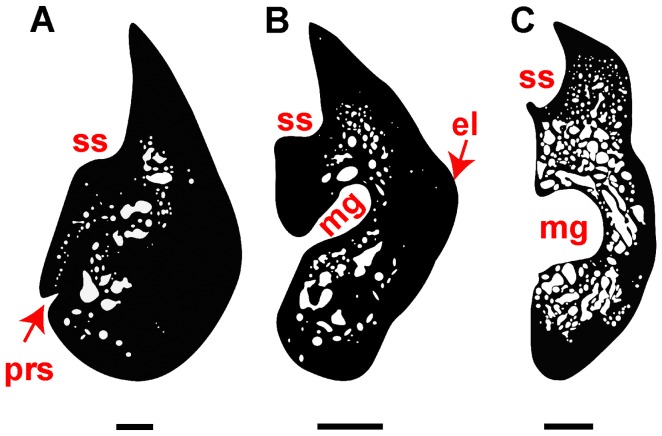
Dentary cross sections of *Aviturus exsecratus*, drawn from natural cross section. (A) position i (PIN 4357/27), (B) pos. ii (PIN 4357/13), and (C) pos. iii (PIN 4357/27). **Abbreviations**: mg, Meckelian groove; el, eminentia longitudinalis; prs, presymphyseal sulcus; ss, subdental shelf. Scale bar = 5 mm.

**Figure 5:**
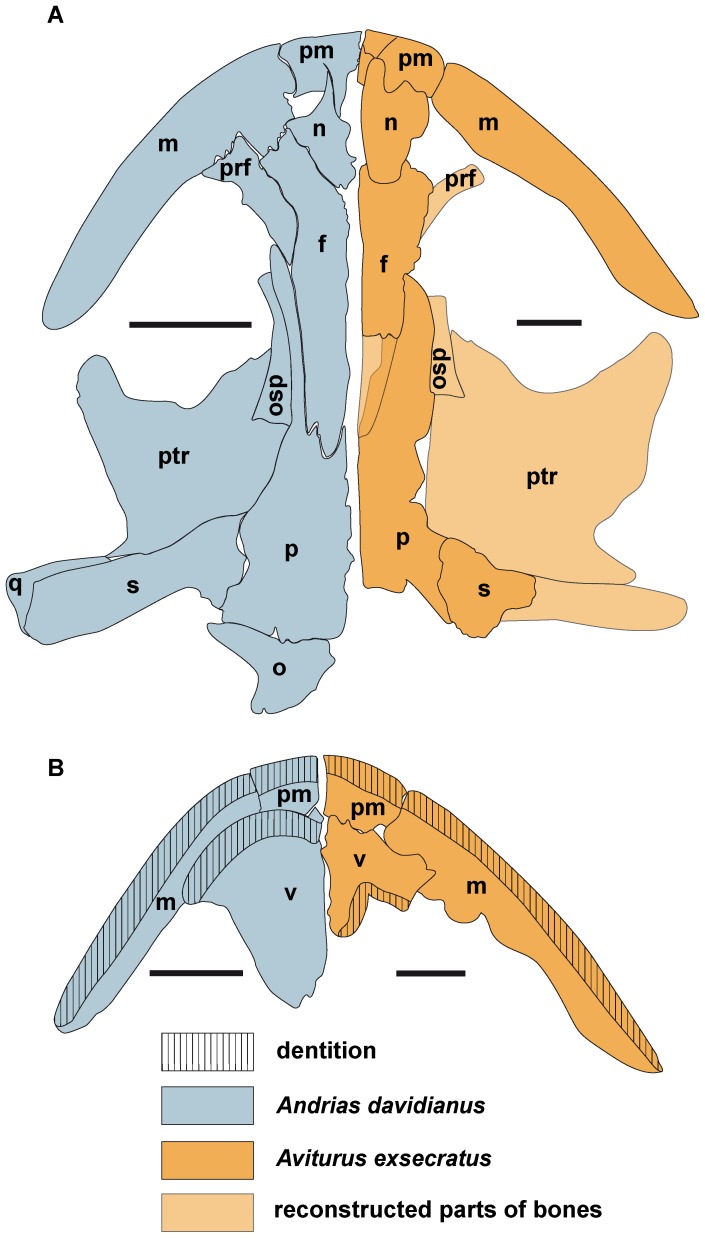
Skull schematic draw of *Andrias davidianus* and skull reconstruction of *Aviturus exsecratus*. (A) in ventral view, showing the dentition pattern on upper jaw and (B) from dorsal view. **Abbreviations**: f, frontal; n, nasal; m, maxillary; o, occipital; p, parietal; osp, orbitosphenoid; pm, premaxillary; prf, prefrontal; ptr, pterygoid; q, quadrate; s, squamosal; v, vomer. Scale bars = 5 mm.

**Figure 6:**
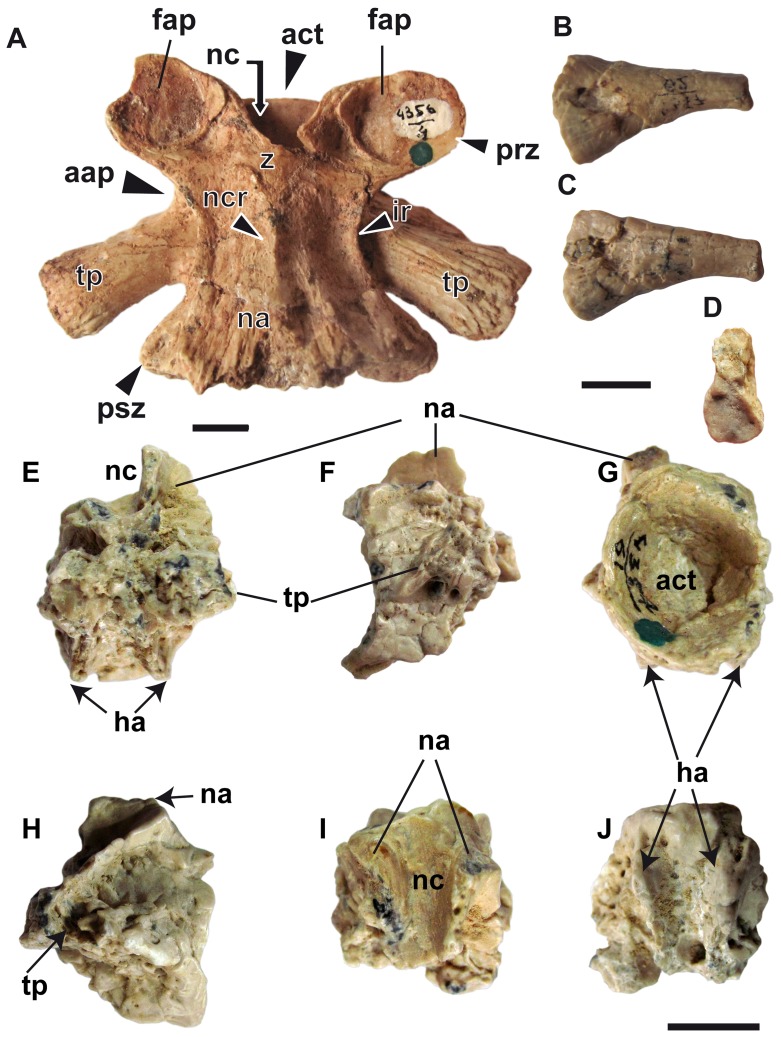
Axial skeleton elements of *Av exsecratus*. (A) PIN 4356/4, a trunk vertebra in dorsal view. (B–D) PIN 4357/62, a rip in anterior (B), posterior (C) and medial (D) views. E–J. PIN 4356/61, a caudal vertebra in posterior (E), left lateral (F), anterior (G), right lateral (H), dorsal (I) and ventral (J) views. **Abbreviations**: aap, anterior alar process; act, anterior cotyle; fap, facies articularis prezygapophysialis; ha, haemapophysis; ir, interzygapophyseal ridge; na, neural arc; nc, neural canal; ncr, neural crest; prz, prezygapophysis; psz, postzygapophysis; tp, transversal process; z, zygosphene. Scale bar = 1 cm.

**Figure 7:**
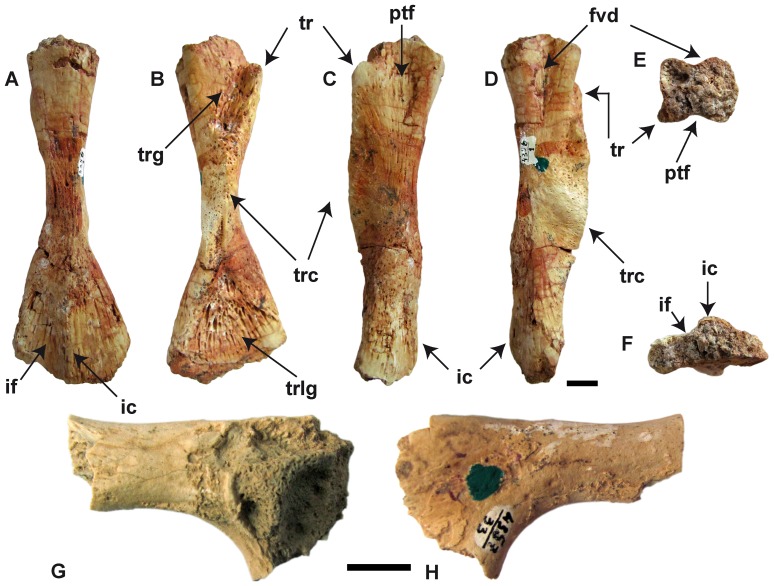
Femur (PIN 4356/1) and ilium (PIN 4357/33) of *Aviturus exsecratus*. Femur in (A) dorsal, (B) ventral, (C) anterior, (D) posterior views and (E) proximal, (F) distal ends. Ilium in (G) lateral and (H) medial sides. **Abbreviations**: fvd, foveal depression; ic, “intercondylar” crest; if, “intercondylar” fossa; ptf, pretrochlear fossa; tr, trochanter; trc, trochanteric crest; trg, trochanteric groove; trlg, trochlear groove. Scale bar = 1 cm.

Fig. 1, 2, 3, 4, 5, 6, 7.

#### Holotype

PIN 4357/27, an almost complete left dentary (Gubin, 1991: fig. 5, tabl.8).

#### Holotype Locality, Age, and Horizon

Aguy-Dats-Bulak locality, Nemengatin Basin, Ömnögovi Province, Mongolia; Naran member of the Naran Bulak formation, cycle VI [9], latest Paleocene, late Gashatan Asian Land Mammal Age.

#### Referred Specimens

Four frontals (two pairs): PIN 4357/11, 15; eight parietals (four pairs): PIN 4357/8–10, 12–14, 73; seven premaxillae (three single, two pairs): PIN 4357/1–6; three maxillae: PIN 4357/21, 22, 24; two nasals (one pair): PIN 4357/7; one quadrate: PIN 4357/19; three squamosals: PIN 4357/8, 12, 73; one occipital: PIN 4357/16; five dentaries (three single, one pair): PIN 4357/27, 28, 32, 4358/1, 2; two vomer: PIN 4357/X; three atlases: PIN 4357/34–36; one femur: PIN 4356/1; one ilium: PIN 4357/33; 13 trunk vertebrae: PIN 4356/2–5, 4357/37, 39, 41, 42, 44, 46, 49, 52, 56; one caudal vertebra: (PIN 4357/61); one rib: PIN 4357/62.

The eight parietals belong to at least four individuals i. PIN 4357/13+73, ii. PIN 4357/08+12, iii. PIN 4357/9+10, iv. PIN 4357/14. The (right) squamosal PIN 4357/73 belongs to the first and the (left) squamosal PIN 4357/12 to the second individual. Two vomers (PIN 4357/X), a right maxilla PIN 4357/21, a pair of premaxillae (PIN 4357/1), a pair of nasals (one pair) (PIN 4357/7) and a pair of frontals (PIN 4357/15) furthermore belong to the i. indivual (see skull reconstruction Fig. 5).

All material coming from the type locality was within a small surface area (1.5×5 m) and belongs to 3–4 individuals. These individuals do not differ significantly from each other and some bones preserve lifetime articulations [8], which we could not be observe by us on the material.

All bones assigned to i. individual originate from the type locality and can be easily assigned to one individual based on the presence of obvious overlapping articulation surfaces and complementing sutures between neighbouring bones. Two (right [PIN 4357/27] and left [PIN 4357/28]) dentaries can be assigned to the i. individual based on their length and the size of reconstructed skull.

#### Distribution

Naran member localities, Nemengatin Basin, Ömnögovi Province, Mongolia [8], [9].

#### Revised Diagnosis

Very large salamanders up to 2 meters, with peramorphic life history strategy and amphibious life style; bones massive and strongly ossified; skull relatively high and triangular in outline; skull roof with a sagittal crest for mandibular levator muscles attachment; vomerine dentition shifted posterior and arranged in a zigzag pattern; long premaxillary facet of the vomer; frontals anteriorly broad; parietals slender; substantially modified large and anteriorly closed cranial cavity with high and strong ossified lateral walls; large and deep olfactory grooves and existing olfactory windows and tracts; parietal-squamosal contact oblique to the sagittal axis; caput squamosa long and wide; posterior squamosal lamina strongly developed; trunk vertebrae with pronounced interzygapophyseal ridge, which covers the proximal part of transverse process; large hind limb (femoral index 2.79); large and long trochanteric crest on femur reaching the trochlear groove, broad and large trochanteric groove, proximal end with pretrochanteric fossa and pronounced foveal depression, dorsal side of distal end with “intercondylar” crest and “intercondylar” fossa, distal and proximal ends of femoral shaft are filled with spongious bone.

### Description

#### Skull

The paired frontals are triangular in outline, anteriorly broad and posteriorly narrow. They are connected to each other along their entire length by a suture. The posterior part of the suture is prominent and forms a median (sagittal) ridge, which widens anteriorly into a triangular plain plateau (Fig. 1). Posterolaterally to this plateau the surface of the frontal is convex and forms a large and elongated facet (Fig. 1) for the attachment of the tendinous sheets of the deep levator mandibulae anterior muscle [10]. The frontals overlie with their posterior portions the parietals and built the parietal ramus. The anterior portion of the frontals is covered with a triangular nasal facet. The lateral positioned facets for the prefrontals are slightly curved and narrow (Fig. 1).

The parietals are also triangular in outline; posteriorly broad and narrowing anteriorly. The right and left parietals are connected along a medial suture along two-third of their length (Fig. 2). The anterior part of the bone, the frontal ramus, is slightly bent laterally and medially not connected to the opposite parietal (there is a deep antero-sagittal slot on the paired parietals). The lateral portion of frontal ramus is plain and belongs to the facet for the attachment of the deep levator mandibulae anterior muscle. The median (sagittal) ridge that originates from the frontal extents onto the parietal, and becomes lower posteriorly (Fig. 2). In this portion, which represents the posterior third of the parietal, the area between the lower medial (sagittal) ridge and the squamosal process is deeply concave for the attachment of the superficial levator mandibulae anterior muscle [11]. The posterolateral corners of parietals are robust and build the squamosal process with a highly rough squamosal facet. The facet is anteromedially directed and has an elongate, oval-shape surface. On the ventral side of each parietal, at the posterolateral corners of the cranial cavity, a relatively large deepening is visible (Fig. 2).

The ventral surface of the parietals and frontals form the roof of the cranial cavity. The cranial cavity of *Av. exsecratus* has an elongate, oval shape, as in recent giant salamanders, and is bordered by the lateral wall (crista sagitalis sensu [12]). The lateral wall has three bumps (posterior, medial and anterior) to connect the cranial cavity roof to the parasphenoid. The posterior and medial bumps lie on the parietals and the anterior one on the frontal. According to the positions of these bumps, we recognize three portions of the cranial cavity (Fig. 1A, 1C). Accordingly, the anterior portion lies below the frontal, the middle portion below both the frontal and parietal, and the posterior one only below the parietal. The anterior portion is nearly heart-shaped and forms the olfactory region of the cranial cavity. Here, as in the recent species [1], lies the forebrain, with its olfactory bulbs. The olfactory region of *Av. exsecratus* is very deep and envelops a large volume. In the anterolateral corners of the cranial cavity, the olfactory windows are developed, from which the olfactory tracts run anterolaterally.

The anterior half of the vomers are preserved (PIN 4357/X). On the smooth dorsal side of the bone an oval nasal process ascends at the anteromedial corner. The ventral side bears a long premaxillar facet in the anterior portion. The dental row shows a dentition pattern that is unusual for cryptobranchids vomers: the tooth row is posteriorly shifted and has a zigzag form (Fig. 3B).

The caput squamosa and the proximal part of quadrate ramus of the squamosals (PIN 4357/12, 73) are preserved (Fig. 3E–G). The bones are relatively flattened and broad. The caput squamosa is long and wide. On its dorsal side, nearly subparallel to the parietal facet, a prominent ridge runs – the marginal lamina. The parietal facet is straight and anteromedially directed (oblique to the sagittal plane), moderately rugose and extents anteriorly into the otic process. A robust, horizontal orientated lamina (posterior squamosal lamina) exists on the posterior border of the proximal quadrate ramus (Fig. 3F). The tendon of the strong anterior depressor mandibulae muscle is attached to this lamina.

The dentary is primarily described by Gubin [8], but additional information can be given. *Av. exsecratus* has, as is typical for cryptobranchids, a convex symphysial contact. We furthermore quantified bone compactness values for the dentary. These value were estimated at the positions i, ii and iii (PIN 4357/13, -/27) (see [5]), and are 0.933, 0.906 and 0.736, respectively (Fig. 4). *Av. exsecratus* shows the highest value of bone ossification among studied giant salamanders. The medullary cavity does not form two large cavities above and under the longitudinal flange. Along the dentary medullary cavity extends and posteriorly strongly defuses in the compact bone.

We reconstruct the outline of the skull based on bones from a single individual (skull length from the snout tip to the end of parietals is ≈18 cm). The bone outlines were drawn from photographs and by comparison to recent giant salamanders. These are: right and left premaxillary (PIN 4357/1), a right maxillary (PIN 4357/21), right and left nasal (PIN 4357/7), right and left frontal (PIN 4357/15), as well as a parietal (PIN 4357/13+73), and the right squamosal (PIN 4357/73), right and left vomer (PIN 4357/X). In our reconstruction the general outline of the skull is nearly triangular (Fig. 5). Since the nasal is relatively broad (Figs. 3C, 3D, 5), the frontal was probably excluded from the naris, like in *Andrias japonicus*, *Andrias davidianus*, and *Andrias scheuchzeri* (Fig. 5).

#### Axial skeleton

The axial skeleton is represented by isolated 17 trunk vertebrae, which are described in Gubin [8]. However, we found some new characters to be described. The vertebrae (PIN 4356/4, 5, etc.) display pronounced interzygapophyseal ridges (Fig. 6A). The accessory alar process is prominent and begins from the anteromedial corner of the transverse process and ends at the ventral surface of prezygapophysis. In some specimens (PIN 4356/2, 3, etc.) the alar process is connected with the interzygapophysal ridge.

The single caudal vertebra is incompletely preserved. Like in other cryptobranchids (but unlike gen. et sp. nov. from the Miocene of Ukraine, see [5]) the bases of the haemapophysis arise in the middle part of centrum (Fig. 6E–J).

The only preserved rib of *Av. exsecratus* (Fig. 6B–D) shows the same morphology as in all other cryptobranchids [5]: an unicapitate articulation surface to the transversal process of trunk vertebra.

#### Appendicular skeleton

The proximal end of the femur (PIN 4356/1) is sub-quadrate in cross-section and has concave anterior (foveal depression) and posterior (pretrochanteric fossa) surfaces (Fig. 7C–E). The trochanteric (femoral) crest is high and massive, and the trochanteric groove is extended. The distal femoral end shows developed “intercondylar” crest and “intercondylar” fossa (Fig. 7A, C, D, F) and expanded trochlear groove (Fig. 7B). The bone is highly ossified.

A single highly damaged ilium (PIN 4357-33) of *Av. exsecratus* is preserved (Figs. 7G, 7H). Earlier the bone was figured but not described in Gubin (1991). The observable morphology of the bone fully agrees with that of recent crpytobranchids. That concerns specially the smooth bone surface, the large acetabulum and distally ascending acetabular surface, the morphology of the ilial shaft as well as that of the medial aspect of the ilium.

## Discussion

**Figure 8:**
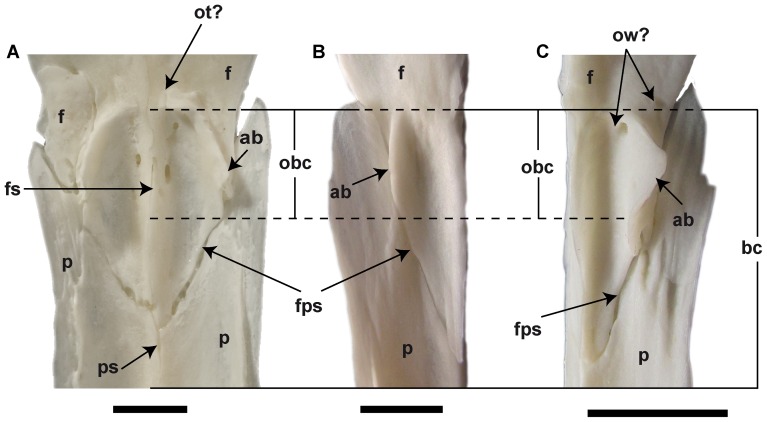
Ventral view of cranial cavity anterior portion in recent cryptobranchids. (A) *Andrias japonicus* (NMA unnumbered specimen). (B) right half of cranial cavity of *An. davidianus* (ZFMK 76996). (C) left half of cranial cavity of *Cryptobranchus alleganiensis* (ZFMK 5245). **Abbreviations**: ab, anterior bump; bc, cranial cavity; f, frontal; fps, frontoparietal sutur; fs, frontal suture; obc, olfactory region of cranial cavity; ot, olfactory tract; ow, olfactory windows; p, parietal; ps, parietal suture. Scale bar = 5 mm.

*Aviturus exsecratus* shows characteristics typical for cryptobranchids, such as the large body size, unicapitate ribs, direct parietal – squamosal connection, as well as bilateral asymmetric kinetics of the lower jaw. The caudal vertebra of *Av. exsecratus* also resembles those of all other giant salamanders, with exception of a new genus from the Miocene of Ukraine. The caudal vertebra of the latter is characterised by elongated oval haemal process, lying in the middle part of the centrum. However, *Av. exsecratus* can be easily distinguished from the recent and other fossil cryptobranchids, by the combination of the following characteristics:

1. *Av. exsecratus* has the most ossified bone tissue among all cryptobranchids. The dentary bone compactness value in *Av. exsecratus* shows the highest values among other giant salamanders [5].
2. The vomerine dentition lies on the ventroposterior surface of vomer and has a zigzag arrangement. All other cryptobranchids (*Zaissanurus beliajevae*(Chkhikvadze, 1982, fig. 4, plate II), *Andrias* spp. (SMNS 7898:1–15, ZFMK 90469, SMNK-PAL.6612), *Cryptobranchus* sp. (ZFMK 5245)) show an anteriorly lying row of vomerine teeth that are oriented parallel to the maxillae and premaxillae.
3. The parietal – squamosal contact is completely different from other cryptobranchids. It is straight and oblique to the sagittal plane in *Av. exsecratus*, whereas in other giant salamanders the contact is slightly bended and runs parallel.
4. *Aviturus exsecratus* has larger and wider caput squamosa.
5. The cranial cavity of *Av. exsecratus* is bordered by prominent lateral walls with three elevated bumps. This lateral wall is poorly developed in the recent giant salamanders, generally it is seen only by the bumps. The olfactory region of the cranial cavity of *Av. exsecratus* is the largest and deepest among cryptobranchids. This region in the recent cryptobranchids is a slightly concave surface or a low deepening, but does not form any groove (Fig. 8). Moreover, the olfactory window and olfactory tract are clearly visible in *Av. exsecratus* (Fig. 1A, C). In the recent species these elements are absent or extremely weak developed (Fig. 8). The anterior end of the cranial cavity is not closed in recent salamanders, whereas in *Av. exsecratus* it is closed.
6. *Aviturus exsecratus* displays the strongest cranial musculature (see below) among cryptobranchids.
7. Unlike other cryptobranchids the trunk vertebrae display strong interzygapophyseal ridges and prominent accessory alar process. Both features indicate strong development of intervertebral muscles, responsible for powerful movements (straightening, stretching, rotating, torsion) of the vertebral column [1].
8. The femur of *Av. exsecratus* can be distinguished from other cryptobranchids by the relatively largest and longest trochanteric crest, which reachs the trochlear groove, whereas in all other giant salamanders the crest terminates in the narrowest section of the bone. The distal and proximal ends of femoral shaft are filled with spongious bone, whereas in all other cryptobranchids both are not filled at all. *Aviturus exsecratus* has the broadest and largest trochanteric groove, which in other cryptobranchids is narrow and shallow, and sometimes not developed at all. The distal part of the bone from the dorsal side shows an “intercondylar” crest and fossa, which are absent in all cryptobranchids. The proximal end of the bone from anterior and posterior view is concave and forms foveal depression and pretrochanteric fossa accordingly. In all other fossil and recent giant salamanders, the foveal depression is slightly visible and the pretrochanteric fossa is absent. Presence of well pronounced, broad and deep grooves and a high crest are evidence for the strongly developed muscular system of the hind limb of *Av. exsecratus* in comparison to other giant salamanders.
9. *Av. exsecratus* has the largest hind limb relative to body size, which was already suggested by Gubin [8]. His observation (relation of length of femur to the length of largest trunk vertebra) was based on comparison of *Av. exsecratus* with only a single specimen of *Andrias japonicus*. We have estimated femoral index of a larger numbers of specimens (Table 1). *Aviturus exsecratus* has the largest femoral index (2.79) among all cryptobranchids (1.83–2.22), although no value can be calculated for *Zaissanurus beliajevae*, which lack associated femur and trunk vertebra from a single specimen.
10. The skull is triangular in outline, whereas in *Andrias* spp. and *Crytobranchus alleganiensis* the skull has an oval form (Fig. 4). Our skull reconstruction differs from one given in Gubin [8]: fig. 1, p. 98, which shows oval form. Unfortunately, we were not able to verify this skull reconstruction, since not all ref. nr. of the bones used in the reconstruction are clear from the text.

**Table 1:** Femoral index (relation of length of femur to the length of largest trunk vertebrae, [8]) in giant salamanders.

Species	n	Femoral index	Specimen or Autor
*Aviturus exsecratus*	1	2.79	Gubin, 1991
*Andrias scheuchzeri*	6	1.82–2.22	SMNK-PAL.6612; SMNK-PAL.6613; PIMUZ A/II 1; PIMUZ A/II 2; [4] *
*Andrias davidianus*	2	1.90–1.96	ZFMK 90469, ZFMK 76996
*Andrias japonicus*	4	1.95–2.24	SMNS 7898:1–15; PIMUZ A 79; [4], [8] *
*Andrias* sp.	2	2.1–2.2	2 unnumbered specimen in NKMB
*Cryptobranchus alleganiensis*	2	1.83–1.92	ZFMK 5245; SMNK unnumbered
Cryptobranchidae gen. et sp. nov., Miocene, Ukraine	1	2.18	[5]

* - For 2 specimen of *An. scheuchzeri* (exemplars in collections of Haarlem and London) and 1 specimen (coll. Darmstadt) of *An. japonicas* the figures in [4] is used to estimate the femoral index.

### Lifestyle and life history strategy of *Aviturus exsecratus*

In general, data on the ontogeny and lifestyle of fossil giant salamanders is scarce [4]. Only for *Av. exsecratus* a non-typical ecology as “an active periaquatic and shoreline predator” was proposed by Gubin [8]: 104 pp., based on peculiarities of the axial skeleton structure, longer limbs, and a higher skull. Our new data confirm this idea. However, due to the detailed analysis of supplementary bones and skeleton parts, e.g., bone ossification, vomer, cranial cavity, as well as skull and axial skeleton, we significantly enlarge our knowledge regarding life style and life history strategy of this oldest Cenozoic cryptobranchid.

#### Vomerine dentition

The vomerine dentition can be classified in regard to its form and position. According to the position, the dental row can be located along the anterior or the posterior side of vomer, whereas according to form it can be arranged in a zigzag or transverse (i.e., parallel to the maxillary and premaxillary dental rows). The anteriorly lying tooth-row of the vomer is defined as the larval-type (non-metamorphic), the posteriorly lying as the adult-type (metamorphic) [13]. So, the posterior located zigzag vomerine dentition type of *Av. exsecratus* is found in several species of hynobiid salamanders [13]. However, within hynobiids there are also different patterns in the vomerine dentition. Whereas the tooth row lies always on the ventroposterior surface of the vomer, the row can either be arranged in a zigzag (e.g., *Hynobius* and *Salamandrella*) or transverse (e.g., *Ranodon* and *Onychodactylus*) [13].

During the metamorphic remodelling of the vomer in recent salamanders (Hynobiidae, Ambystomidae, Dicamptodontidae, etc.) the larval-type tooth-row is replaced by an adult-type tooth-row [14]. So far, this remodelling was not found in recent or in fossil cryptobranchids.

The zigzag arrangement of the vomerine dentition is characteristic of “pond-type” salamanders [13], in which larval development undergoes in still water and the adult feeding is via tongue protraction. “Pond type” salamanders live in humid lowlands and have a terrestrial lifestyle. The transverse form of tooth row on vomer characterises “stream-type” salamanders [13], in which larval development undergoes in flowing, running water. The adults feed by prehension or suction and live and feed underwater. Transversely oriented vomerine teeth may hinder escape of prey when water is released from the mouth, whereas the “pond-type” species mainly feed on small terrestrial invertebrates, which they capture with tongue movements that deliver the prey deep within the mouth, where they are held by the posteriorly directed (zigzag shaped) vomerine tooth row. Similar types of vomerine teeth are present, besides in hynobiids, in plethodontid and salamandrid salamanders, which use the tongue for prey capture [13]. Based on these observations, *Av. exsecratus* is characterized by a peramorphic life history strategy, “pond-type” vomerine dentition and amphibious lifestyle.

#### Terrestrial feeding

*Aviturus exsecratus* has, as typical for cryptobranchids, a convex symphysial contact, which produces a highly mobile mandibular symphysis with two pads of elastic cartilages. The smaller dorsal and larger ventral pads fill triangular spaces between the dentaries and are compressible [11]. This allows *Aviturus exsecratus* to move the lower jaw during feeding bilaterally asymmetric [11], which provides increased mobility and better manipulation during prey capturing under water.

The morphological investigation of the frontals, parietals, and squamosals reveals a strong development of the mandibular levator and depressor muscles, suggesting substantial force during prey capturing [5], [11]. In accordance to increased biting force, the connections of the skull bones are strongly developed, especially the vomerine-premaxillary and the parietal-squamosal contacts. Combining the vomerine dentition type with these lines of evidence, we may suggest that beside the ability of underwater feeding *Aviturus* also possessed a terrestrial mode of feeding.

#### Olfactory region

*Aviturus exsecratus* is characterised by a well differentiated olfactory region, olfactory tract, and olfactory window of the cranial cavity (Figs. 1A and 1C) in comparison to recent giant salamanders (Fig. 8). For comparison with recent giant salamanders we used both young (skull length 6–8 cm, see Figure 8) and adult individuals (skull length 11 cm [*Andrias davidianus* ZFMK 90469] and 15.5 cm [*Andrias japonicus* PIMUZ A 79]). Both young and adult individuals do not show different degree of developmental of olfactory region of cranial cavity. So, no ontogenetic changes for this character could be observed on the available material. Two existing *Aviturus exsecratus* frontals (PIN 4357/11, 15) are of similar size and also show similar morphology of the olfactory region.

Differentiation of an olfactory lobe, which is located in olfactory region of the cranial cavity, in the anterior part of the cerebral hemisphere occurs in caecilians and anurans [1]. It is poorly developed in most salamanders and is absent in obligate neotenes [1]. The degree of development of the olfactory lobe is positively correlated with development of Jacobson's organ, which is absent in paedomorphic salamanders. There is considerable variation in the complexity of the olfactory organ among salamanders. It is least complex in aquatic taxa, and tends to be more complex in more terrestrial species [1]. The olfactory sense is more important for orientation and seeking prey in terrestrial animals. The well pronounced olfactory region of *Av. exsecratus* is therefore further evidence of its terrestrial lifestyle and non-paedomorphic life history strategy.

#### Skeleton (bone ossification and femur)

Further evidence for a terrestrial lifestyle of *Aviturus exsecratus* is apparent in the skeleton. *Av. exsecratus* has the highest degree of ossification skeleton among giant salamanders, which characterize terrestrial adults in amphibians [15]. As evidenced by the femur, *Av. exsecratus* has the relatively longest hind limbs and well developed process and surfaces for muscle attachment (e.g., trochanteric crest, “intercondylar” crest, “intercondylar” fossa, see Figure 7). These characteristics, including the general femur morphology, are terrestrial adaptations resembling the late Palaeozoic genus *Eryops*, a temnospondyl amphibian. Recent results interpret the lifestyle of *Eryops* either as more terrestrial [16], or more amphibious [15], [17]. So, according to Pawley and Warren [16] *Eryops* displays following terrestrial adaptations: highly ossified bones, comparatively large limbs, and well-developed processes for muscle attachments. Whereas, Schoch [15] and Witzmann [17] interpreted the highest degree of ossification of bones, as well as much larger arms and legs as evidences for semi-terrestrial (amphibious) life style of *Eryops*.

#### Sedimentologic and taphonomic indications

All described *Av. exsecratus* fossils derived from terrestrial, pedogenized overbank sediments [9], [18]. These sediments formed the top of alluvial cycles typical for meandering river systems under seasonal climates; starting with cross-stratified sands and ending with white-red mottled palaeosols. None of bones show signs of abrasion. All bones were found as associated, partly articulate skeletons over an area of 1.5×5 meters [18], indicating no post-mortem transport of individual skeleton parts. The palaeosols don't contain any aquatic fossil (e.g., fishes, which are found frequently in the sands). This stratinomic, taphonomic, and lithologic observations point to the suggestion that the burial place in overbank soils near a stream is similar to the living habitat of *Aviturus*.

### Conclusions

#### Metamorphosis and Cryptobranchidae

**Figure 9:**
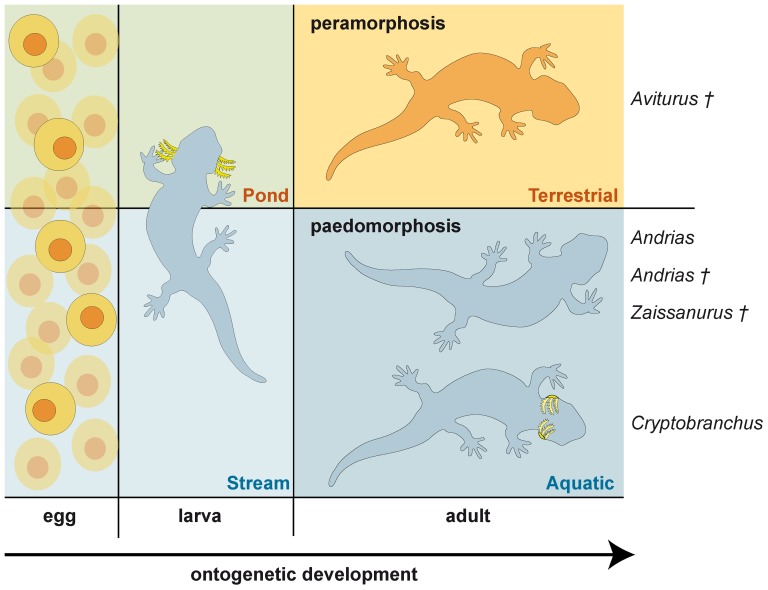
Ontogenetic diversity within Cryptobranchidae.

The three recent cryptobranchids species are strictly aquatic and obligate paedomorphic salamanders. According to our data, the fossil taxa *Andrias scheuchzeri* and *Zaissanurus beliajevae* were also aquatic and paedomorphic. Notable, the recent *Cryptobranchus alleganiensis* is the most paedomorphic, whereas as our studies (data in prep.) showed that recent and fossil species of *Andrias* and *Z. beliajevae* have the same level of paedomorphic development (Fig. 9).

Obligatory paedomorphosis, where the trait is fixed genetically, often at higher taxonomic levels, is associated frequently with environmental adaptation to large, generally permanent bodies of water, including large streams, rivers, lakes, and swamps, as seen in Amphiumidae, Cryptobranchidae, Sirenidae, and (in part) Proteidae [19]. Obligatory paedomorphosis represents an adaptive route to exploiting permanent aquatic habitats in harsh terrestrial environments, including subterranean environments [19]. However, all salamanders excluding cryptobranchids are able to complete their metamorphosis under certain environmental and/or physiological conditions [19], whereas giant salamanders do not undergo metamorphosis even by hormonal treatment [14].

The presence of metamorphic characteristics in *Aviturus* does not necessarily indicate metamorphosis during their ontogeny (i.e., a drastic, short phase of rapid changes, in which a water-living larva transforms into a small-sized land-living adult; see [15]), but rather a peramorphic developmental pattern (Fig. 9). Peramorphosis is the opposite to the pattern of paedomorphosis, during which amphibians create a terrestrial morphology by extending life span, i.e., large intervals between ontogenetic events – resulting in enlargement of body size under permanent ontogenetic development [15]. So far, peramorphic developmental patterns are exclusively known from the early amphibian eryopines [15]. Our results from *Av. exsecratus* indicate for the first time pronounced peramorphosis within a crown-group lissamphibian. Strongly pronounced peramorphic characters within the vomerine dentition, olfactory region, vertebrae morphology, and bone ossification makes *Av. exsecratus* unique among all lissamphibians. Moreover, *Aviturus exsecratus* is not only a cryptobranchid with peramorphic characters, but also the first giant salamander with partially terrestrial (amphibious) lifestyle. Accordingly, its morphology shows the ability both underwater and terrestrial feeding modes.

#### *Aviturus exsecratus* and the Paleocene–Eocene Thermal Maximum

The *Av. exsecratus* materials derive from the upper part of the Naran Member within Naran Bulak Formation in South-Central Mongolia [9], [18], [20]. The Naran Member can be correlated to the late Gashatan Asian Land Mammal Age and the late Clarkforkian North American Land Mammal Age [21]–[23]. The *Aviturus*-bearing horizons lying only few meters below the base of the earliest Eocene Bumban Member, thus chronostratigraphically very near to the Paleocene–Eocene Thermal Maximum (PETM; [24]). The PETM is characterized by a transient global temperature rise, probably accompanied by a rise in atmospheric CO_2_ [25]. The response of continental ecosystems to this hypothermal event is still poorly known, however, it has been proposed that North American mammals reacted by transient dwarfing [26]. Whether cryptobranchids responded to the PETM by a shift in their ontogenetic trajectories is speculative, in particular so, because high-resolved chronologic dates are missing from the Naran Member. Future investigations are needed to understand the response of amphibian ecology to rapidly increased atmospheric temperatures and CO_2_ levels. According to the phylogenetic analysis of Vasilyan et al. [5] *Aviturus exsecratus* is the stratigraphically oldest member of the family Cryptobranchidae. The appearance of cryptobranchids at the Paleocene–Eocene boundary parallels that of several mammalian orders [22], [26], which substantiate this time as the main Cenozoic turnover event in terrestrial ecosystems.

## Materials and Methods

Anatomical terminology follows Vasilyan et al. [5]. Some of osteological terms are new.

### Institutional Abbreviations

NMA, Naturmuseum Augsburg, Augsburg, Germany; PIMUZ, Palaontologisches Institut un Museum der Universitat Zurich; PIN, Paleontological Institute, Russian Academy of Sciences, Moscow, Russia; SMNK, Staatliches Museum für Naturkunde, Karlsruhe, Germany; SMNS, Staatliches Museum für Naturkunde, Stuttgart, Germany; ZFMK, Zoologisches Forschungsmuseum Koenig, Bonn, Germany.
